# Expression of claudin-5, claudin-7 and occludin in oral squamous cell carcinoma and their clinico-pathological significance

**DOI:** 10.4317/jced.52801

**Published:** 2016-07-01

**Authors:** Ekarat Phattarataratip, Kraisorn Sappayatosok

**Affiliations:** 1 DDS, PhD, Assistant Professor. Department of Oral Pathology, Faculty of Dentistry, Chulalongkorn University, Bangkok, Thailand; 2 DDS, PhD, Assistant Professor. Faculty of Dental Medicine, Rangsit University, Thailand

## Abstract

**Background:**

Claudin and occludin are the important tight junctions protein in human. The downregulation or upregulation of claudins and occludin might have a role in cancer development. The objective of this study was to investigate the expression of claudin-5, claudin-7 and occludin in oral squamous cell carcinoma (OSCC) and their relationships with the prognostically-related clinico-pathologic features.

**Material and Methods:**

Standard indirect immunohistochemical technique using anti-claudin-5, anti-claudin-7 and anti-occludin was performed in formalin-fixed paraffin-embedded tissue sections of 66 OSCC samples from Faculty of Dentistry, Chulalongkorn University. The positive cases were divided into 2 groups, the low expression group (cases with less than 50% of positive cancer cells) and the high expression group for statistical analysis. Categorical analysis of the clinico-pathologic parameters together with univariate analysis using the Kaplan-Meier method and the log rank test were performed.

**Results:**

There were 22 male and 23 female patients enrolled in this study, with a mean age of 65.82+12.10 years. The claudin-5 immunoreactivity was observed in 26.6% of cases. The positive immunoreactivity of claudin-7 is more noted (93.3%). Only 4 cases showed occludin immunoreactivity (8.9%) and all of them show positivity less than 25% of cancer cells. Only loss of claudin-7 expression was associated with the high pathologic grade, advanced TNM staging, large tumor size, the presence of microscopic perineural, vascular invasions and regional lymph node involvement. There is a tendency towards the association of the higher claudin-7 expression and a longer survival time (*P*=0.012).

**Conclusions:**

The results showed expression of claudin-5, claudin-7 and low expression of occludin in OSCC. Only claudin-7 expression showed impact on clinic-pathological parameter of OSCC.

** Key words:**Claudin, occludin, oral squamous cell carcinoma, tight junctions, oral cancer.

## Introduction

Intercellular junctions are important structures for physiologic functions of the cells. Tight junctions (TJs), one of the intercellular junctions, play a main role for signaling cascades that control cell growth and differentiation (1). TJs are thought to play critical roles in the neoplastic process from their roles in extracellular and intracellular signaling pathways (2).

TJ comprise of three major transmembrane proteins, namely claudins, occludin, and junctional adhesion molecules (3). Currently, at least 24 different members of the claudin family are known in humans. They act as a barriers for cellular ionic selectivity seen in epithelium. Expression of claudins may vary in different cells and tissues of the body. For example, claudin 2 is found in murine liver and kidney (4) but not in lung tissue while claudin 4 is found in murine lung and kidney but not in the liver (5).

Abnormalities of TJ permeability allow increased diffusion of factors that promote tumor growth. Moreover, changes in TJs have been noted as an early event in tumor metastasis (6). Downregulation or upregulation of claudins might have a role in cancer development. The altered expression of some claudins has also been found in many human carcinomas such as those of the oral (7), breast (8), ovary (9), and stomach (10).

Claudin expression has been also shown to have prognostic value in many tumors. Loss or gain in claudin expression has been associated with biologic behavior in some tumor types (11-13). Reduced expression of claudin will result in cell to cell adhesive-ness breakdown which can promote cancer invasion and metastasis. However, the mechanism by which overexpression of claudins may contribute to tumor progression and aggressiveness is less clear. It has been suggested that a possible mechanism for this is that up-regulation and/or aberrant tissue expression of claudins may directly interfere with TJ formation and function and thereby contribute to neoplasia. Nevertheless, relatively few studies have described the expression of claudins or their relationship with tumor activity or behavior in oral squamous cell carcinoma (OSCC) (3,8,13).

Occludin expression decreased progressively in parallel with the increase in carcinoma grade, and the decreased occludin expression correlated with myometrial invasion and lymph node metastasis in endometrial carcinoma (14). About squamous cell carcinoma, there are only few studies showing expression or clinical correlation of occludin staining (13,15).

No study, to the best of our knowledge shows the association of claudin-5, claudin-7 together with occludin expression in oral squamous cell carcinoma (OSCC) and their clinic-pathological prognostic factors. Therefore, the objective of this study was to investigate the expression of these two members of the claudin family and occludin in OSCC and their relationships with the prognostically-related clinical-pathologic features.

## Material and Methods

Forty five of oral squamous cell carcinoma (OSCC), from various sites in the oral cavity in the archive of biopsy specimen at the Department of Oral Pathology, Faculty of Dentistry, Chulalongkorn University since 2006-2012 were recruited for the study. Re-examination of histopathologic slides were examined under light microscope by two oral pathologists, both to confirm the diagnosis and grading of tumor. Briefly, the tissue sections were cut at 4-µm thick, initially stained with hematoxylin and eosin (H&E). The patients’ history were reviewed. Other clinical data such as patients age, gender, lymph node status and recurrent were collected. Cases were excluded if the specimen included any other associated pathology (e.g. chronic fungal, bacterial infection or other tumors).

For immunohistochemical study, procedure was performed with Leica Microsystems Bond-Max Autostainer System. Paraffin-embedded blocks from the tumor with adjacent areas and control tissue specimens were 5-µm thick then deparaffinised with Bond Dewax Solution. The antigen retrieval was performed by incubating slides with the Bond Epitope Retrieval Solution 2 for 30 minutes at 95oC for claudin-5 and 7 staining. For occludin staining, the section was treated with pronase (Tris-HCl, pH 7.6) for 8 minutes at 37°C and then cooled for 3 minutes at 4°C.

The primary antibodies used were the polyclonal anti-claudin-5 (1:200 dilution), monoclonal anti-claudin-7 (1:500 dilution) anti-bodies (Invitrogen, Camarillo, CA) and polyclonal anti-occludin antibody (1:50 dilution).

A 3-step indirect immunoperoxidase technique was performed using the Bond Polymer Refine Detection kit (Leica Microsystems). The primary antibodies were incubated for 50 minutes at room temperature, then 3% hydrogen peroxide was applied for 5 minutes, followed by 12 minutes incubations with the Post Primary Polymer and the Polymer Poly-HRP IgG, respectively. The sections were reacted with diaminobenzidine chromogen for 3 minutes and counterstained with hematoxylin. The Bond Wash Solution was used to rinse between each step. The colon mucosa were used as positive controls. Negative control staining was carried out by substituting nonimmune rabbit or mouse serum and phosphate-buffered saline (PBS) for the primary antibodies.

-Immunostaining assessment and statistical analysis

The immunohistochemical assessment was performed and agreed upon by two pathologists who were blinded to all patient clinical and follow-up data. The positive immunoreactivity localized at the cell membrane of neoplastic cells was evaluated. Overall, the percentage of positive neoplastic cells was semi-quantitatively assessed and categorized into one of the following groups: 0 = no positive cells; 1+ = positive cells detected less than 25% of tumor; 2+ = positive cells detected between 26-50% of tumor; 3+ = positive cells detected between 51-75% of tumor and 4+ = positive cells detected more than 75%. The expressions of claudin-5, claudin-7 and occludin were further classified as low immunoreactivity (groups 0, 1 and 2) and high immunoreactivity (groups 3 and 4) for statistical analysis.

The results were statistically analysed using the IBM SPSS Statistics version 21 (IBM Corporation, NY) for Windows. The con-tinuous variables were expressed as means + standard deviation (SD). Categorical analysis of the clinico-pathologic parameters and the claudin-5, claudin-7 and occludin expressions were performed using either the Pearson’s chi-square test or the Fisher’s exact test, as appropriate. A *P*-value less than 0.05 was considered statistically significant.

For analytical statistical analysis, the clinico-pathologic features were grouped as following: age below or above 65 year-old (the mean age of patients); tumor size (T1-T2 and T2-T4); early TNM stage (I, II) or late TNM stages (III, IV); microscopically well differentiated or moderately/poorly differentiated; the presence of perineural invasion and vascular invasion, local recurrence, regional lymph node involvement and distant metastasis.

The disease-specific survival was determined as the time following surgical operation to the time of patient died of cancer. Univariate analysis using the Kaplan-Meier method and the log rank test were used to analyze the significant differences between groups.

The study was ethically approved by ethical committee, Faculty of Dentistry, Chulalongkorn University, Thailand. This study was conducted in strict accordance with the ethical principles of the Declaration of Helsinki.

## Results

-Patient characteristics

Clinical and pathological detail of the patients are shown in  Table 1. There were 22 male and 23 female patients enrolled in this study, with a mean age of 65.82+12.10 years (range= 44-86 years). Most of lesions were located on gingiva (37.8%), followed by floor of mouth (17.8%), tongue (15.6%) and buccal mucosa (13.3%). Recurrence was found in ten patients (22.2%). Twenty-four patients (53.3%) had regional lymph node involvement, and 2 of them had distant metastasis (4.4%). The majority of patients were classified as TNM stage I (40.0%), followed by stage IV (31.1%). 53.3% of cases was well-differentiated squamous cell carcinoma, followed by moderately differentiated (33.3%) and poorly differentiated (13.3%). Perineural invasion was found in 17 patients (37.8%) while perivascular invasion was found in 19 patients (42.2%).

**Table 1:**
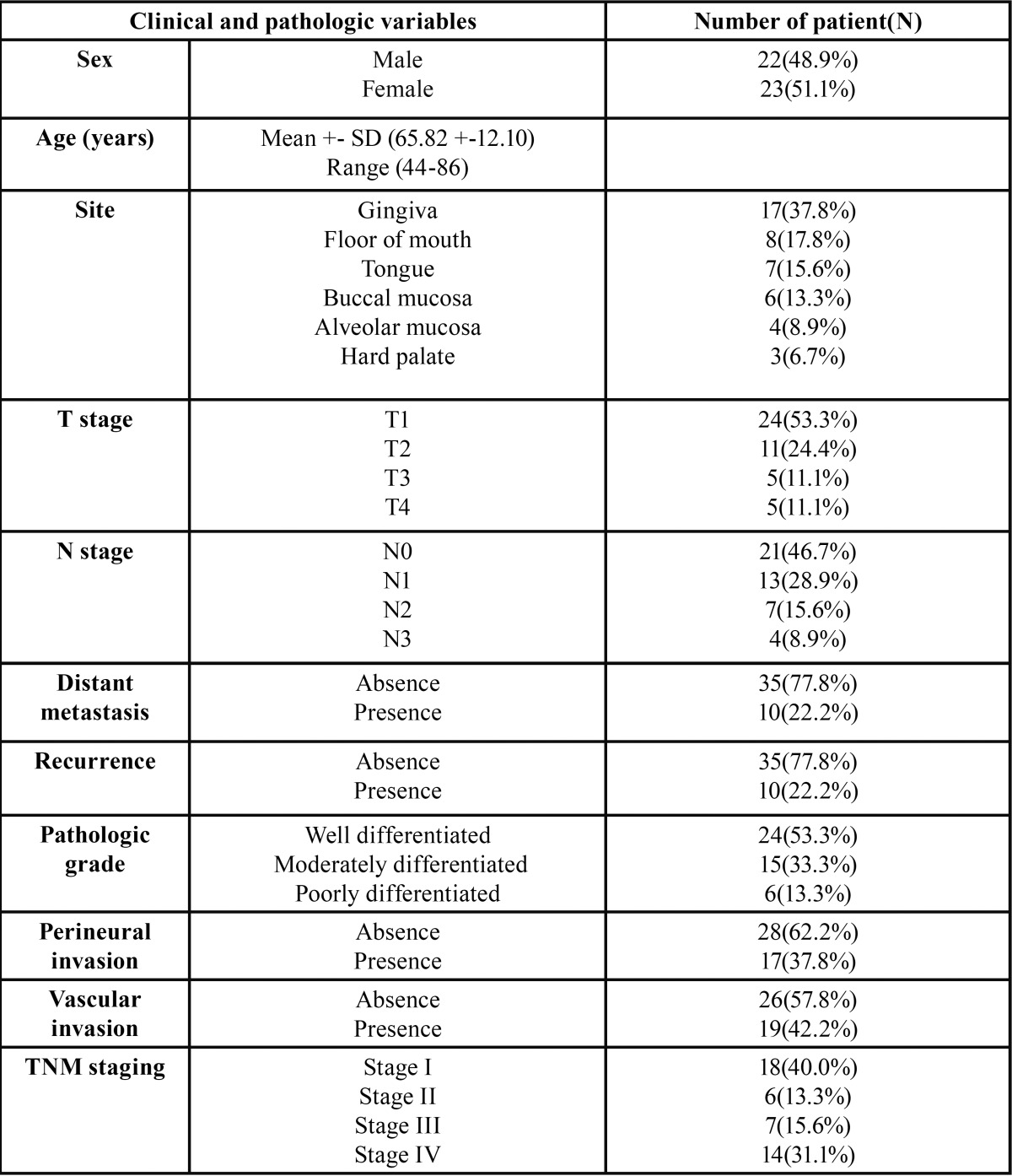
Clinical and pathological detail of the patients.

-Expression of claudin-5 and claudin-7 in OSCC

**Figure 1:**
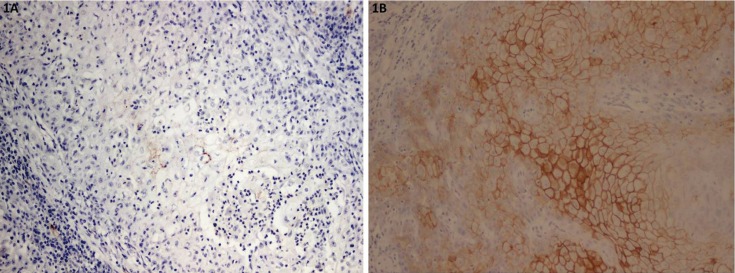
Representative photomicrographs of mild staining of claudin-5 (1A) and intense staining of claudin-5 (1B).

**Figure 2:**
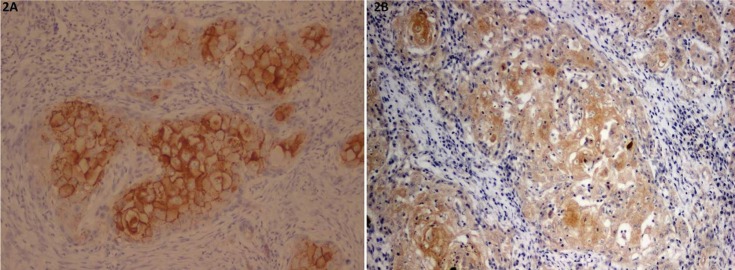
Representative photomicrographs of plasma membrane staining of claudin-7 (2A) and intense plasma membrane and cytoplasmic staining of claudin-7 (2B).

**Figure 3:**
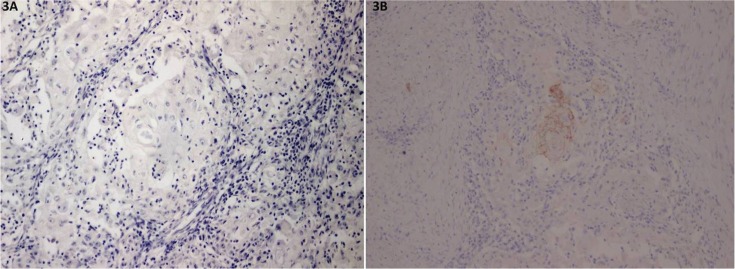
Representative photomicrographs of negative staining of occludin (3A) and mild staining of occludin (3B).

Immunohistochemical staining of OSCC cells was shown in figures 1,2,3 and  Table 2. The tumor cells in the central area shows intense staining than cells in peripheral area. All positive cells for claudin-5 and occludin show cell membrane staining pattern except claudin-7 which also shows cytoplasmic staining. The claudin-5 immunoreactivity was observed in 26.6% of cases. The majority of them (17.8%) showed positive staining less than 25% of cancer cells (level 1+; 17.8%), followed by the staining in 51-75% of cells (level 3+; 6.7%) (Fig. 1). The positive immunoreactivity of claudin-7 is more noted (93.3%). Twenty-five cases (55.5%) showed claudin-7 positivity more than 50% of cancer cells in which 20 cases (44.4%) showed positivity more than 75% (Fig. 2).

**Table 2:**
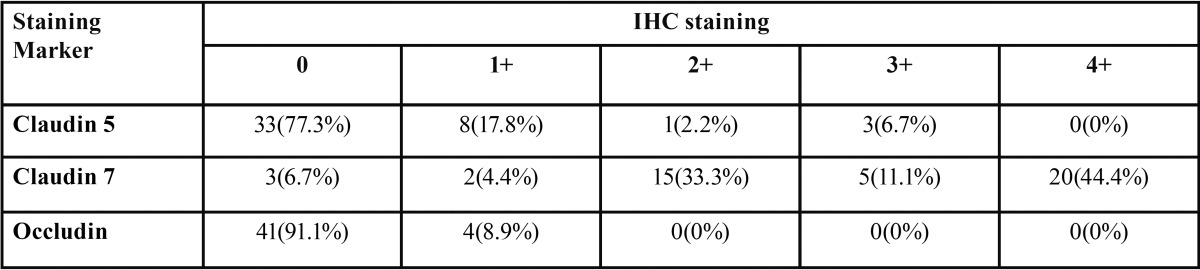
Immunohistochemical staining of OSCC cells.

While only 4 cases showed occludin immunoreactivity (8.9%) and all of them show positivity less than 25% of cancer cells (Fig. 3).

-Relationships between the claudin-5 and claudin-7 expressions and the clinico-pathologic features

The cases positive to claudin-5 and claudin-7 were divided into 2 groups, the low expression group (cases with less than 50% of positive cancer cells) and the high expression group (cases with more than 50% of positive cancer cells) for clinicopathological correlation. No significant found between occludin expression and clinicopathological parameters due to low number of cases positive to occludin.

Relationship between claudin-5 and claudin-7 expression and clinico-pathologic features of OSCC patients are shown in  Table 3. No sex or age difference was observed between the two groups. Significantly, loss of claudin-7 expression was associated with the high pathologic grade (*P*=0.027), advanced TNM staging (*P*=0.00), large tumor size (*P*=0.01), the presence of microscopic peri-neural (*P*=0.01) and vascular (*P*=0.031) invasions and regional lymph node involvement (*P*=0.001).

**Table 3:**
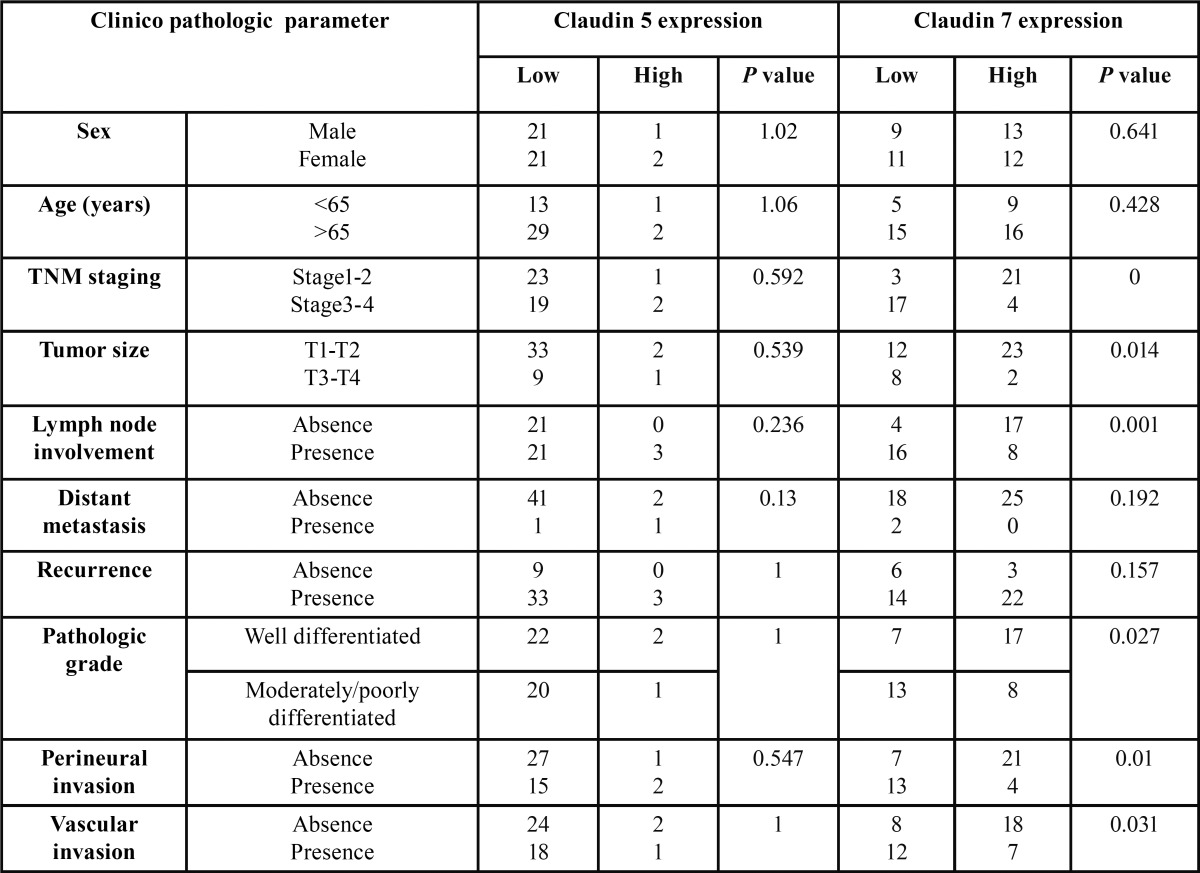
Categorical analysis of the clinico-pathologic parameters and the claudin-5, claudin-7 expressions using either the Pearson’s chi-square test or the Fisher’s exact test, as appropriate.

-Survival analysis

**Figure 4:**
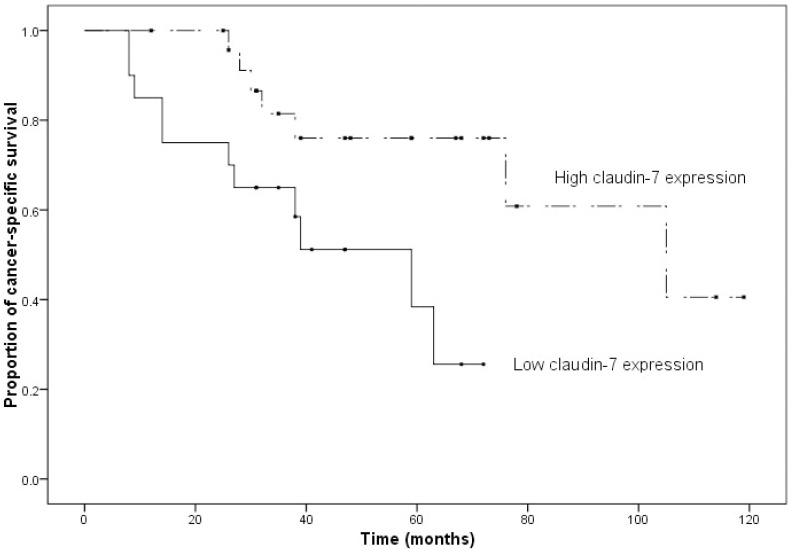
Kaplan–Meier curve of OSCC patients with low (less than 50 % positivity) versus high (more than 50 % positivity) expression of claudin-7 (*P* = 0.012)

The follow up period ranged from 8 to 119 months (median=38 months). Seventeen patients died of OSCC at the end of the follow-up period. Two patients died of other causes and the remaining 26 patients were alive with no disease. The advanced clinical staging was strongly correlated with the poor overall patient survival (*P*=0.01). Regarding claudin expressions, the univariate survival analysis showed that claudin-5 expression has no statistically significant association with cancer-specific survival of patients (*P*=0.131) while there is a tendency towards the association of the higher claudin-7 expression and a longer survival time (*P*=0.012) (Fig. 4).

## Discussion

There are several studies demonstrated expression of different tight junction proteins in a variety of human cancers (7,16). Among these tight proteins junction, claudin and occludin are main important molecules .

The dissociation of cancer cells from the primary cancer nests is a crucial step for cancer progression and metastasis. Loss of cell-cell adhesiveness may trigger the release of cancer cells from the primary cancer nests and confer invasive properties on a tumor (2).

Claudin 7 expression was found to be down-regulated in ductal carcinoma of the breast cancer (17), cervical cancer compared with CIN/CIS lesions (18) , and squamous cell carcinoma of esophagus (19). In contrary, increased expression of claudin 7 has been also reported in gastric adenocarcinomas (20) invasive carcinoma of the uterine cervix (21). The results for the discrepancy are still not well understood.

In our study, we reported the potential role of claudin-7 in clinico-pathological correlation of OSCC. Loss of claudin-7 expression was associated with the high pathologic grade advanced TNM staging, large tumor size, the presence of microscopic perineural and vascular invasions and regional lymph node involvement. These findings are consistent with previous reports of Melchers that lack of claudin-7 expression in the tumor center may be used to identify patients with increased risk for regional recurrence of OSCC (22). The study of Usami (19) in esophageal cancer also shows that expression of claudin-7 at the invasive front is statistically correlated with the depth of invasion, stage, lymphatic vessel invasion, and lymph node metastasis.

Also, higher claudin-7 expression in our study relates with longer survival time which seems that claudin 7 expression may be a favorable prognostic significance in patients with OSCC like studies in many other centers by (11) (13,23). The study in hepatoce-llular carcinoma by Bouchagier also shows that down-regulation of claudin-7 are positive prognostic markers and are associated with good outcome and increased survival rates (24). This prognostic implication was not shown in occludin or claudin 5.

Nevertheless, in some studies high claudin-7 expression was significantly associated with a poorer prognosis of the patients with gastric cancer (20) which did not correspond with our study. Also the study in urothelial carcinoma shows no clinicopathological correlation of claudin-7 expression (25).

Claudin-5 expression is revealed to correlated with lymph node metastasis in esophageal carcinoma (12) which is not shown in our study. Down-regulated claudin-5 expression in tumor vessels may serve as a potential marker for poor prognosis in hepato-cellular carcinoma (26) while increased claudin-5 expression is associated with aggressive behavior in serous ovarian adeno-carcinoma (27). The reason why expression of claudin-5 in many cancer is different is still a controversial issue.

Occludin is believed to be not essential for TJ formation and function but may play a role in cellular signaling. The expression of occludin in our study is mostly negative which corresponds with study in tongue squamous cell carcinoma (13). While study in hepatocellular carcinoma (24), urothelial carcinoma show occludin expression without clinic-pathological impact (25).

Loss of claudin expression of the cell can directly promote the neoplastic process because the TJs were destroyed. Moreover, lack of claudin-7 or less tight junctions consequently increased availability of nutrients and ligands (28). This would lead to in-creased proliferation, in agreement with study of Ding showing the increase proliferation of epithelial cells in the intestine of claudin-7 deficient mice (29).

Conversely, there are some experiments showing that overexpression of some claudins may interfere with the functional tight junctions, instead, overexpression of them can reversely increased tumor invasion. The mechanism by which overexpression of claudin may contribute to tumor progression is less understood. Study by Lioni in *in vitro* knockdown of claudin-7 resulted in increased invasion in esophageal SCC (30). Other mechanism proposed may involve increased matrix metalloproteinase 2 activity by signaling cascade of claudin.

## Conclusions

Based on this study, there are claudin-5 and 7 expression in OSCC. Only claudin-7 expression might be useful for predicting OSCC behavior and prognosis of the patients.
